# The Supression of Migration and Metastasis via Inhibition of Vascular Endothelial Growth Factor in Pancreatic Adenocarcinoma Cells Applied Danusertib

**DOI:** 10.5152/tjg.2024.22319

**Published:** 2024-02-01

**Authors:** Erkan Alabaş, Ahmet Ata Özçimen

**Affiliations:** Department of Biology, Mersin University Faculty of Science, Mersin, Turkey

**Keywords:** Aurora kinase, CFPAC-1, danusertib, metastasis, migration, vascular endothelial growth factor (VEGF)

## Abstract

**Background/Aims:**

Pancreatic ductal adenocarcinoma is an extremely deadly type of cancer with a high metastatic potential. Genetic factors in cellular events play an important role in the emergence of this situation. One of these factors is Aurora kinase family members, which play a role in migration, invasion, and cell cycle. In this study, the expression of vascular endothelial growth factor gene, which plays a role in migration, metastasis, and angiogenesis, on cystic fibrosis human pancreatic ductal adenocarcinoma 1 cells of danusertib, a pan-Aurora kinase inhibitor, was examined.

**Materials and Methods:**

The half maximal inhibitory concentration (IC_50_) value (400 nM) of danusertib in cystic fibrosis human pancreatic ductal adenocarcinoma 1 cells was determined by the wound-healing test depending on the dose and time and migration with CIM-Plate 16 in the xCELLingence system. In addition, the effect of danusertib on migration was determined by quantitative reverse transcription polymerase chain reaction (qRT-PCR) method and vascular endothelial growth factor gene expression.

**Results:**

When the dose- and time-dependent danusertib-applied cystic fibrosis human pancreatic ductal adenocarcinoma 1 cells were compared with the control group, it was observed that the wound formed did not close. In the xCELLigence system CIM-Plate 16 migration analysis, it was observed that migration was inhibited in the group administered danusertib in parallel with the wound dehiscence experiment. The gene expressions of vascular endothelial growth factor decreased 0.5-fold at the 24th hour and 0.3-fold at the 48th hour in the Danusertib-administered groups.

**Conclusion:**

Danusertib, a pan-Aurora kinase inhibitor, is predicted to be used as a potential agent in pancreatic cancers due to its anti-tumor and anti-metastatic effect.

**Main Points**

- In the study, the effects of danusertib have been investigated in highly metastatic pancreatic adenocarcinoma cells. According to the results of the research,

- Danusertib has arrested migration by using xCELLigence real-time cell migration analysis;
- Danusertib has shown the inhibition effect of the metastasis by using wound-healing assay;
- Danusertib has shown the diminishing effect of vascular endothelial growth factor gene expression in these cells.

## Introduction

Pancreatic cancer includes both exocrine and endocrine tumors of the pancreas. More than 90% of pancreatic tumors arise from the ductal epithelium. Pancreatic ductal adenocarcinoma (PDAC) is an extremely aggressive malignancy with a high metastatic potential.^1,2^ Increase in the overall survival rate for pancreatic cancer is weak, and due to aggressive metastatic spread, the vast majority of patients die. This highlights the need to develop new therapeutics that target not only the primary tumor but also the biological weaknesses of metastatic pancreatic cancer cells.^2^ Pancreatic ductal adenocarcinoma represents the fourth leading cause of cancer death worldwide and the 12th most common cancer in the World.^3^ Cancer is a highly complex process involving genetic and epigenetic changes that result in the activation of oncogenic pathways and/or inactivation of tumor-suppressor signals. During cancer progression, cancer cells acquire a number of distinctive features that promote tumor growth and spread.^4^ Cancer cells that trigger proliferation, suppress growth and enable them to escape from apoptosis signals; it acquires malignancy through molecular changes that promote angiogenesis, invasion and metastasis.^5^ In addition, most cancer-related deaths are caused by distant metastases not the primary tumor. Therefore, the ability of cancer cells to migrate and enter surrounding tissues and distant organs is another important sign of cancer.^6^ Cancer progression is greater than changes in cancer cells because the variability in the tumor microenvironment plays a critical role in tumor development and progression as well as drug efficacy.^7^ Cancer cells have a unique ability to adapt to different environmental conditions by acquiring different morphologies and migration characteristics to remain mobile.^8,9^ Cell migration and invasion are important steps in many physiological events such as embryogenesis, angiogenesis, wound healing, and inflammation.^10,11^ These steps play a role in the pathophysiology of many diseases such as cancer. The state of spread of tumor cells, which is very different among cancers, is the main feature of malignant tumors and is one of the main causes of death for cancer. This is because metastases consist of cells that are much more resistant and aggressive than the cells that make up the primary tumor.^12^ However, when the cancer has spread to a different location from the primary site, they are often incurable and fatal. Most cancer deaths are caused by cancer metastasis and not the primary tumor.^13^ Cancer cells not only grow in the tissue where they are located but also multiply in other tissues by metastasizing.^14,15^ Metastasis is the main cause of treatment failure for cancer patients.^15^ Metastasis is a multistep process in which cancer cells leave the primary tumor niche and invade other sites in the body.^16,17^ Metastasis development requires cancer cells to leave their primary site, enter the bloodstream, withstand the pressure in the blood vessels, acclimate to the new cellular environment at a secondary site, and escape immune cells.^18^ Metastasis accounts for more than 90% of cancer-related mortality. At the metastatic site, cancer cells initiate colonization and often use preexisting blood vessels to form new tumors.^19^ Pancreatic cancer is a malignancy with a tendency for local spread and distal metastasis. Although largely non-vascular, these cancers exhibit foci of micro-angiogenesis and overexpress multiple pro-angiogenic factors.^20,21^ Although the specific mechanisms of pancreatic cancer have not been clearly established, it is characterized by the overexpression of mitogenic and angiogenic growth factors and their receptors as well as various molecular changes.^21^ Vascular endothelial growth factor (VEGF), one of these growth factors, plays an important role in the development of pancreatic cancer.^22^ Vascular endothelial growth factor, an endothelial cell mitogen, induces angiogenesis.^23^ Vascular endothelial growth factor expression and low lymphatic vessel density can be considered as malignant prognostic factors because tumors with this profile multiply rapidly.^24,25^ Vascular endothelial growth factor is also overexpressed in human PDAC.^26,27^ Vascular endothelial growth factor is expressed in most tumors, and its expression correlates with abnormal growth of tumor cells.^28^ Therefore, cancer drugs have an important place in cellular events. Due to the therapeutic inhibition of kinases that have essential roles during cell division, potential anticancer therapy studies have increased significantly. Important kinase inhibitors of the cell cycle include Bcr-Abl tyrosine kinase and Aurora kinases.

Aurora kinases are an important family of protein kinases that are cell cycle regulators. Aurora kinases consist of 3 members (Aurora A, Aurora B, and Aurora C) and are involved in mitotic events, chromosome segregation, cytokinesis, and cellular events such as meiosis.^29^ Expression of Aurora A is very low during the G1 phase and begins to accumulate in the centrosome in the late S phase, with a maximum at the G2/M transition. During this period, it is localized at the spindle poles and degrades before cytokinesis.^30^ Aurora B is a critical chromosomal transition protein for chromosomal segregation, cytokinesis, regulation of microtubule–kinetochore attachments toward the centrosome and kinetochore, and regulation of the mitotic checkpoint.^31^ Aurora B begins in early G2 and locates on chromosomes in prophase, centromeres in prometaphase and metaphase, central spindle in anaphase, and mid stem in cytokinesis.^32,33^ Aurora C localizes to the centrosome during mitosis from anaphase to cytokinesis and plays a role in centrosome function at a later stage of mitosis.^34^ The overexpression of Aurora kinases in human cancers and their importance in controlling the mitotic process in cell division has led to the development of small-molecule inhibitors as anticancer drugs.

Based on the pan-Aurora kinase inhibitor danusertib, whose antitumoral activities were determined in human pancreatic adenocarcinoma cells in previous studies, it was predicted in this study to be a potential antimigratory and antiangiogenic agent.^35^

Danusertib is a third-generation Bcr-Abl tyrosine kinase inhibitor with Aurora kinase and potent anticancer activity. Danusertib produces an increase in the expression of pro-apoptotic proteins but together with a decrease in antiapoptotic proteins causes mitochondria-mediated apoptosis.^36^ Danusertib is the most advanced clinical compound that potently inhibits all Aurora kinase family members with predominant inhibition of Aurora kinase B. Danusertib has therapeutic potential with anticancer effects in a wide variety of cancers, including solid tumors and leukemias.^37^ Danusertib is a small-molecule 3-aminopyrazole derivative with potent activity against Aurora kinases. Danusertib is a pyrollo-pyrazole that inhibits Aurora kinase A, B, and C, with IC_50_s of 13, 79, and 61 nmol/L, respectively, and Abl tyrosine kinase of 25 nmol/L. Based on these data, danusertib can be identified as a spectrum-selective kinase inhibitor for cancer-related kinases.^38^ Studies have shown that the Aurora kinase family is effective for pancreatic cancer.^39^

Therefore, in the planned study, it was aimed to investigate the anticancer effect of danusertib on cystic fibrosis human pancreatic ductal adenocarcinoma 1 (CFPAC-1) cells.

Cystic fibrosis (CF) is a chronic inherited disease that damages the electrolyte transport properties of epithelial cells in the sweat glands, pancreas, and other organs. Cystic fibrosis human pancreatic ductal adenocarcinoma 1 cells are a pancreatic adenocarcinoma cell line derived from a patient with CF. The cells show epithelial morphology as well as cytokeratin and oncofetal antigen characteristics of pancreatic duct cells.^40^

In conclusion, the aim of this study is important in terms of guiding future in vivo drug research by focusing on migration, metastasis, and gene expression of danusertib, which has an anticancer effect in human PDAC cells.

## Materials and Methods

**Figure 1.**
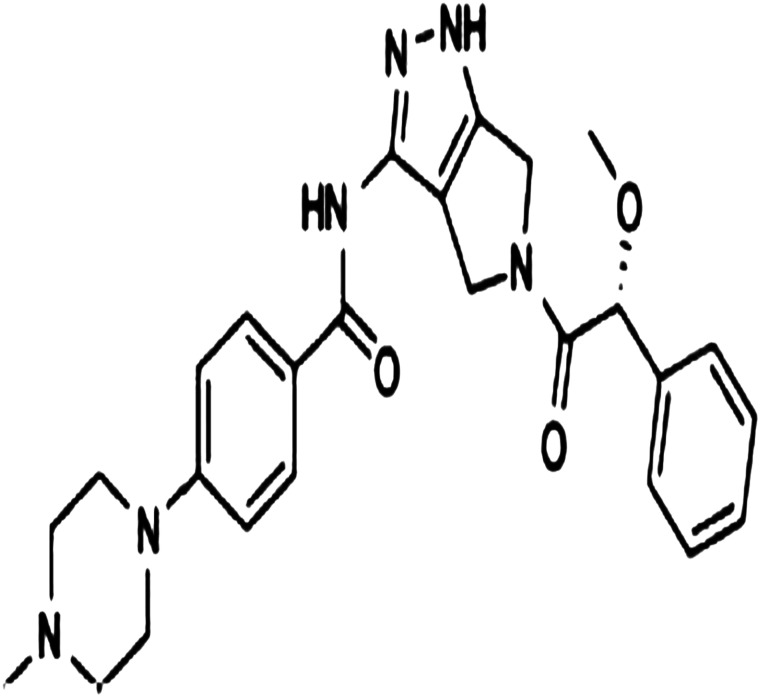
Chemical structure of danusertib.^36^

Danusertib (PHA-739358) (Figure 1) 10 mg was obtained from Mybiosource (San Diego, Calif, USA). Fetal bovine serum (FBS), trypsin, penicillin, streptomycin, amphotericin B, Dulbecco’s phosphate buffered saline (DPBS), l-glutamine, and Iscove’s modified Dulbecco’s media (IMDM) were purchased from Biowest (Bradenton, Florida, USA). Gotaq PCR Master Mix was purchased from Promega (Wisconsin, USA). The CIM-Plate 16 system was purchased from ACEA Biosciences (San Diego, Calif, USA). Beta actin and VEGF gene primers were purchased from Macrogen (Seoul, South Korea). Transcriptor First Strand cDNA Synthesis Kit and High Pure RNA Isolation Kit were purchased from Roche (Germany).

### Cell Culture

Cystic fibrosis human pancreatic ductal adenocarcinoma cells were cultured in IMDM medium containing 10% FBS, 1% l-glutamine, 1% penicillin + streptomycin, and 1% amphotericin-B. The cells were grown in the optimum conditions of 37°C, 95% humidity, and 5% CO_2_. All experiments were performed with the same passage number of cells and the same conditions.

### Drug Application

In a previous study, the cytotoxic IC_50_ dose of danusertib in CFPAC-1 was found to be 400 nM.^35^ Their activity on these cells will again be carried out over the known effective cytotoxic IC_50_ dose.

### Demonstration of Migration by Wound-Healing Assay

The migration of cells was demonstrated by the wound-healing assay.^41^ For the wound-healing experiment, CFPAC-1 cells were seeded (5 × 10^5^ cells per well) into 6-well plates (Corning, Sigma-Aldrich, Burlington, Massachusetts, USA). Cells incubated for 24 hours or more were viewed to be confluent. It was incubated for 24 hours with a medium without FBS. Then, the medium in the wells was removed, and parallel wounds were opened with a pipette tip in a straight line. After the cells that were washed 3 times with DPBS were removed from the medium, a medium with 1% FBS and 400 nM Danusertib was added. Photographs of the IC_50_-dosed cells were taken at certain time intervals (0, 18, 24, 48, 72, and 96 hours). As for wound closure, the groups were compared between the control and each other.

### Migration Assay by xCELLigence System

Migration experiment was carried out with CIM-Plate 16 application in xCELLigence device with IC_50_ dosage determined by obtaining from the drug at all time intervals up to 0-99 hours. Cystic fibrosis human pancreatic ductal adenocarcinoma 1 cells were taken into a serum-free medium the night before. About 160 μL of medium IMDM with 20% serum was put into the lower cup of CIM-Plate 16. In the upper part, 90 μL of a serum-free medium was put into each well and kept at 37°C for 60 minutes. One hour later, the first step (1 minute, first sweep) was read. Cells were washed with DPBS during a 1-hour dwell time. Cells were removed by trypsinization. Cystic fibrosis human pancreatic ductal adenocarcinoma cells were diluted with a serum-free medium, and 20 000 cells were added to the upper of the CIM-Plate 16 at 110 µL (100 µL +10 µL drug). CIM-Plate 16 was incubated for 30 minutes at room temperature, and cells were observed to descend to the bottom. CIM-Plate 16 was inserted into the instrument and measured using the xCELLigence RTCA (ACEA Biosciences, Calif, USA) system in 15-minute cycles for up to 99 hours.

### Total RNA Isolation

To measure the gene expression, total RNA was purified to extract RNA from the CFPAC-1 cell line using the High Pure RNA Isolation Kit (Roche, Germany). Total RNA was obtained from danusertib-treated (400 nM) and untreated CFPAC-1 cells depending on dose and time (24 hours and 48 hours).

### Reverse Transcription Quantitative Reverse Transcription Polymerase Chain Reaction

Total RNA for RT-PCR was isolated from CFPAC-1 cells. Complementary DNAs were synthesized by taking 1 µg from total RNA using the High Capacity cDNA Reverse Transcription kit (Applied Biosystems, Thermo Fisher Scientific, Waltham, MA, USA).

Complementary DNA synthesis (Techne Thermal Cycler, Bibby Scientific, Staffordshire, UK) was performed using the High Capacity cDNA Reverse Transcription kit (Applied Biosystems, Thermo Fisher Scientific from total RNAs obtained from CFPAC-1 cells. cDNAs were loaded in appropriate groups into the ViiA 7 qRT-PCR device (Applied Biosystems, Thermo Fisher Scientific). For the expression level of VEGF gene expression, 40 cycles of amplification were performed using VEGF Sybr Green gene primers and reference gene Beta actin (housekeeping) primers. Quantitative analysis was performed with 2^−ΔΔCT^ values in accordance with the Pfaffl method.

### Statistical Analysis

Migration in this study was statistically determined by the RTCA software in the xCELLigence system and the cell index analysis program. Migration results were evaluated using xCELLigence RTCA software (ACEA Biosciences, San Diego, Calif, USA). Vascular endothelial growth factor gene expression level was calculated using the 2^−ΔΔCT^ formula appropriate according to the Pfaffl method. Data analysis was evaluated by the ΔΔCT method and quantified with a computer program called “ViiA 7 qRT-PCR Software” (Applied Biosystems, Thermo Fisher Scientific). Statistical analyses of gene expressions and activities were performed via the Web-based QuantStudioTM Software V1.2.4.

## Results

### Danusertib Suppresses the Motility and Migration of Pancreatic Adenocarcinoma Cells In Vitro

**Figure 2.**
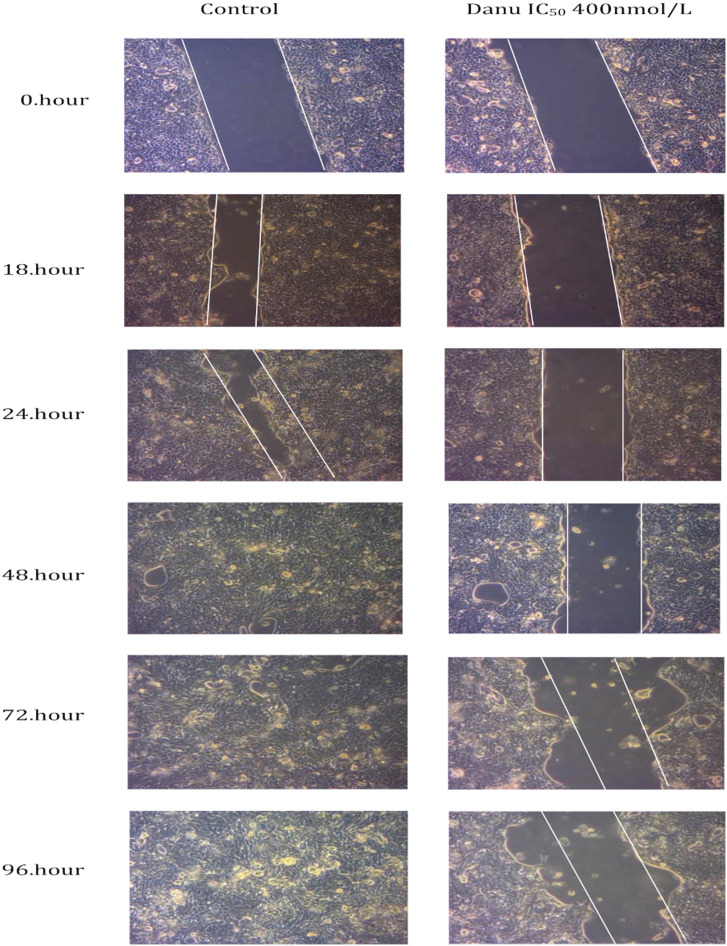
Danusertib suppressed cell migration as determined by the wound-healing assay. The images of the wounds of the control and danusertib 400 nM/L-applied pancreatic adenocarcinoma cells with a pipette tip at 0 hours were photographed with a microscope until the 96th hour. Visualization of the wound closure distance at the wound site (compared to the control at *t* = 0) was observed (at ×400 magnification).

It is known that migration and invasion are the main features of tumor metastases.^41^ The wound-healing assay was performed to test whether danusertib had an inhibitory effect on migration. After the addition of the dose danusertib to the cells, streak-shaped wounds were opened. Migration of cells to the wound area was followed for certain hours (0-96 hours). In the non-Danusertib control group, CFPAC-1 cells migrated to the no-cell area (Figure 2). However, danusertib 400 nM dose inhibited the migration of cells in the experimental group, since there was no migration to the area of the wound.

### Effect of Danusertib on Migration of Pancreatic Adenocarcinoma Cells Cystic Fibrosis Human Pancreatic Ductal Adenocarcinoma 1

**Figure 3.**
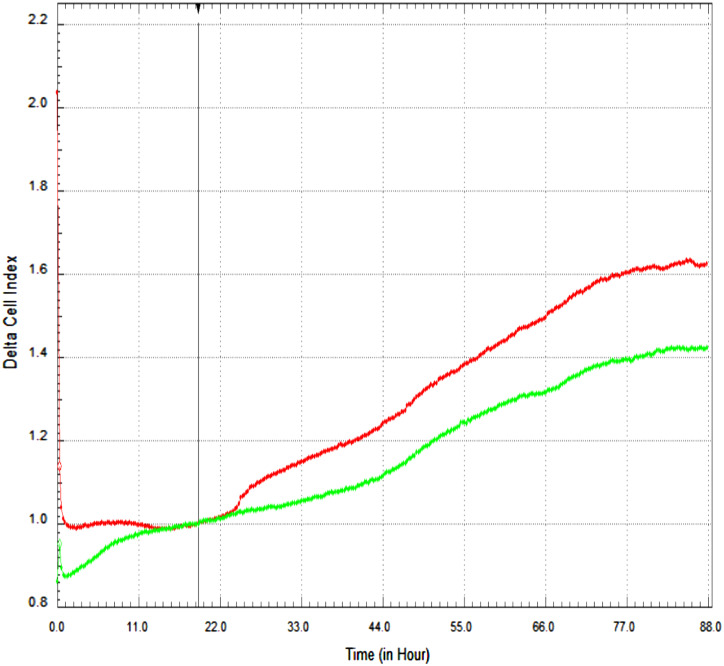
CIM-Plate 16 migration analysis result of CFPAC-1 cells in the xCELLigence system. Red: control; green; danusertib IC_50_ refers to the 400 nM group. About 160 μL of medium was added to the lower part of the CIM-Plate 16 and 110 μL (100 μL cells + 10 μL drug) was added to the upper part. CIM-Plate 16 was incubated for 30 minutes at room temperature. CIM-Plate 16 was placed on the device and measurements were made in 15-minute cycles for up to 99 hours.

The xCELLigence RTCA system was used to measure the effect of danusertib IC_50_ value on migration. In the region indicated by the black vertical line, delta normalization was performed for the cell populations from the software, and the growth curves were overlapped. In the graph of the CIM-Plate 16 migration analysis result in the xCELLigence system, no migration was observed in CFPAC-1 cells that were administered danusertib 400 nM dose (Figure 3).

### The Effect of Danusertib on Vascular Endothelial Growth Factor Gene Expression, Which Plays an Important Role in the Metastasis Mechanism in Cystic Fibrosis Human Pancreatic Ductal Adenocarcinoma 1 Cells

**Figure 4.**
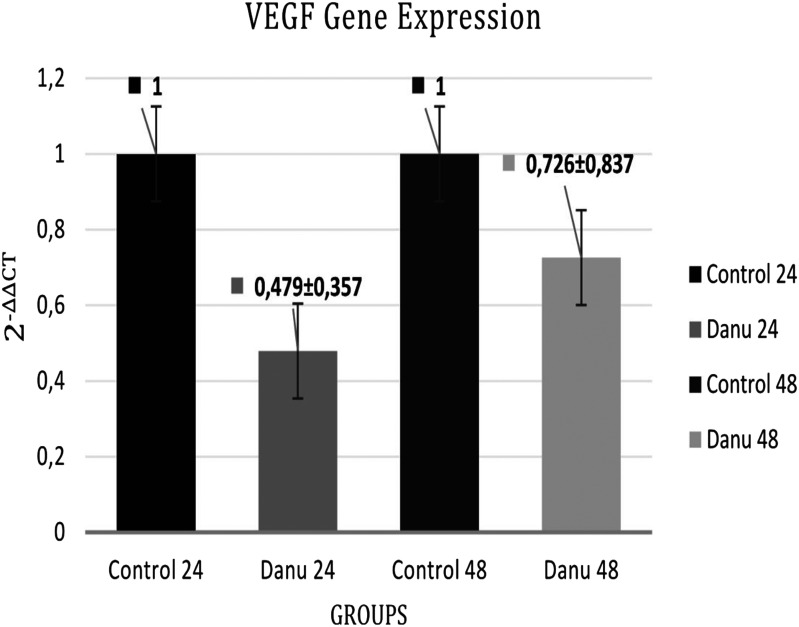
Danusertib reduces VEGF gene expression levels in migration and metastasis. Results were presented as means ± SD of at least triplicate experiments; each condition was performed with 3 cultures.

The primer sequences used for gene expression in qRT-PCR are given in Table 1. Vascular endothelial growth factor gene expression in CFPAC-1 cells with and without administration of danusertib was quantitatively measured using the ViiA 7 qRT-PCR device, depending on the dose and time (24 hours and 48 hours). There was viewed decrease a 0.479 ± 0.357 fold change in VEGF expressions decreased by 0.479 ± 0.357 times at the 24th hour drug group and 0.726 ± 0.837 times at the 48th hour drug group (Figure 4). It is observed that the effect of the drug on VEGF gene expression level in CFPAC-1 cells is greater at the 24th hour but less at the 48th hour. This shows that the danusertib drug used is effective in the first 24 hours, and its effect decreases toward the following hours. In conclusion, the decrease in VEGF gene expression shows that angiogenesis, which plays an important role in the metastasis mechanism, is suppressed.

## Discussion

Cancer invasion and metastasis are important events that transform a locally growing tumor into a metastatic and threatening disease. In the last decade, cell and tumor biologists have described cell migration mechanisms in normal and malignant cells.^8^ The sensitivity of cell migration, its mechanism of action, the time, speed, direction and control target of migrating cells are important in studying the molecular mechanisms of cancer.^10^ Tumor growth is dependent on angiogenesis by the changes brought about by microvessels in solid tumors.^20^ Angiogenesis is a critical step in the progression of nearly all human malignancies and some life-threatening diseases. Antiangiogenic therapy is a new and effective approach in the treatment of angiogenesis-related diseases such as cancer.^19^ Inhibitors affecting the function of kinases in biological research are valuable for the treatment of kinase-related diseases such as different cancers.^29,42^ Among mitotic regulatory kinases, the evolutionarily conserved family of serine/threonine kinases termed “Aurora kinases” emerged as an extremely attractive target for anticancer drug discovery. Overexpression of the Aurora kinase family in solid tumors is among the important causes of the increase in cancer.^32,34^ There are many different small-molecule inhibitors developed for Aurora kinases.^42^ These kinase inhibitors have been found to have antitumor effects against different cell types such as hepatocellular carcinoma Hep3G cells, chronic myeloid leukemia cell lines, and mammary gland MCF7 and MDA-MB-231 cells.^43-45^ Aurora kinase inhibitors have shown intriguing efficacy in human tumors. Among these, one of the currently most advanced clinical compounds is danusertib, which acts as an inhibitor against all known Aurora kinases as well as other cancer-associated kinases such as Bcr-Abl tyrosine kinase.^46^ Danusertib was used as a pan-Aurora kinase inhibitor in this study. The studies have shown a significant anticancer and antimetastatic effect of Danusertib in gastric, melanoma, ovarian carcinoma, leukemia, and different cancer cell lines.^36-38^

In this study, danusertib showed antimigratory and antiangiogenic effects by suppressing the VEGF gene, which plays a role in angiogenesis, as well as by inhibiting migration on CFPAC-1. In our results, danusertib has been viewed to be effective in CFPAC-1 cells at 400 nmol/L. The effects of danusertib on the kidney are seen at high-dose exposure.^47^ The dose of danusertib (400 nmol/L) used shows a greater effect on cancer cell types, unlike healthy cells, suggesting that it will yield more positive results in anticancer drug treatments.

The failure in the treatment of pancreatic carcinoma is mainly caused by the metastasis of tumor cells to neighboring organs.^48^ Migration is an important event in tumor metastasis. This situation is clarified by our data in Figures 2 and 3 in our study. The wound-healing assay was performed to evaluate the motility of CFPAC-1 cells. As a result of this experiment, we observed that danusertib significantly inhibited migration in pancreatic adenocarcinoma cells in a dose- and time-dependent manner. In the study by Xie and Meykens,^37^ it was shown that danusertib, which has antiproliferative and antimetastatic effects, has an effect on migration in SK Mel28 melanoma cells at low danusertib dose (100 nM). Some growth factors are also effective in the migration of tumor cells. Vascular endothelial growth factor plays an important role in the inhibition of cancer metastasis as an effective factor in events such as angiogenesis, migration, and wound healing. In a study, VEGF expression, which is seen as a mitogenic factor, was shown in human pancreatic cancer cell lines (COLO-357, MIA PaCa-2, PANC-1, T3M4, ASPC-1, and CAPAN-1).^26^ Vascular endothelial growth factor is a potent angiogenic growth factor. It is overexpressed in numerous malignancies, including breast, colorectal, and liver carcinoma.^49,50^ Overexpression of VEGF and its receptors provide the survival advantage and regulation of tumor growth.^20^ In the study, time/dose depended Danusertib in CFPAC-1 cells was shown that VEGF gene expression was decreased 0.5-fold at 24th hour and 0.3-fold at 48th hour. Hence Danusertib might be crucial role in metastasis of pancreatic adenocarcinoma (CFPAC- 1).

Overall, Danusertib appears to have a potential role and antimetastatic effect to inhibit the in-vitro migration of aggressive migrant pancreatic adenocarcinoma cells. It is thought that Danusertib may play an important role in future studies on cancer metastasis. Nevertheless, the research on the effects of Danusertib should be supported in conjunction with several in vitro methods. Thus, the preliminary study will shed light on future research in the in vivo studies.

In conclusion, pan-Aurora kinase inhibitor danusertib may be a potential agent with antitumor and antimetastatic effects in pancreatic cancers. However, it is predicted to support the study by conducting further in vivo studies.

## Figures and Tables

**Table 1.** Primer Sequences Used for Quantitative PCR

Gene	Primers
VEGF	Forward: 5′-CTACCTCCACCATGCCAAGT-3′
	Reverse: 5′-GCAGTAGCTGCGCTGATAGA-3′
Beta actin	Forward: 5′-GAGGTGATAGCATTGCTTTCG-3′
	Reverse: 5′-CAAGTCAGTGTACAGGTAAGC-3′

VEGF, vascular endothelial growth factor.
